# Diagnostic Yield of Delayed Phase Imaging in CT Angiography of the Head and Neck: A Retrospective Study

**DOI:** 10.1371/journal.pone.0099020

**Published:** 2014-06-06

**Authors:** Debbie L. Bennett, Leena M. Hamberg, Bing Wang, Joshua A. Hirsch, R. Gilberto González, George J. Hunter

**Affiliations:** 1 Massachusetts General Hospital, Department of Radiology, Boston, Massachusetts, United States of America; 2 Brigham and Women's Hospital, Department of Radiology, Boston, Massachusetts, United States of America; 3 The University of Texas MD Anderson Cancer Center, Diagnostic Radiology, Houston, Texas, United States of America; University Medical Center (UMC) Utrecht, Netherlands

## Abstract

**Purpose:**

To evaluate how often delayed images, obtained during neurovascular CTA, provide unique information relative to early phase imaging alone.

**Materials and Methods:**

Informed consent was waived by the institutional review body for this study. Neurovascular CTAs from January through June 2009 were searched to identify those with delayed phase imaging. Reports were reviewed to identify cases where delayed images provided potentially unique information. The studies with potentially unique information were re-interpreted to determine if the information was indeed unique.

**Results:**

645 CTAs with delayed phase imaging were identified. There were 324 men and 310 women (median age 67 years; range 20–96 years). 59 studies (59/645: 9.1%) had findings on the delayed images. There were 13 cases with hemorrhage, with 4 showing progression on delayed views. Of the remaining 46 cases, 28 had occlusion of a vessel that did not reconstitute on the delayed images, 6 had occlusion of a vessel that did reconstitute on the delayed images, 7 had a string sign which was unchanged on the delayed views and 5 had no abnormal findings. Thus in 10 cases the findings were unique to the delayed images (10/645: 1.55%). Four showed active bleeding, three showed proximal occlusion with distal internal carotid filling from ophthalmic collaterals, two showed pial vessels filling distal to proximal MCA occlusion, and one showed retrograde vertebral artery filling due to subclavian steal. 95% confidence limits of the expected incidence of unique information from the delayed phase images are 0.6% – 2.5%.

**Conclusion:**

Obtaining delayed phase imaging for neurovascular CTA should be an active decision and not the default protocol. This avoids imaging with little, if any value. If delayed images had not been obtained in our cohort, no detriment in patient management would have occurred.

## Introduction

The component of neurovascular CTA that is primarily focused on the intracranial circulation is used to identify intracranial hemorrhage (ICH), hemorrhagic or ischemic stroke, aneurysm, and vasculitis [1], [2]. It is also used to identify the location and potential involvement of vascular structures that could affect surgery. For example, the presence of anomalous venous anatomy could alter the approach to posterior fossa surgery, or invasion of vascular structures could change the surgical approach when performing tumor resection [3]–[6]. Extending a head CTA to include the neck vasculature allows evaluation of the vascular effects of cranio-cervical trauma, carotid or vertebral artery stenosis, traumatic or spontaneous vessel dissection and fibromuscular dysplasia [7]–[12]. Further extending coverage to include the aortic arch allows identification of great vessel stenosis, aortic dissection, the distribution of plaque burden in the aorta, and proximal common carotid or vertebral artery stenosis at the thoracic inlet [7], [13].

Indications for neurovascular CTA can be grouped into two broad categories: (a) emergency evaluation because of suspected acute stroke, trauma affecting the head, neck or great vessels, or potential intracranial hemorrhage [14]–[17] and (b) non-emergency evaluation of the degree of vessel stenosis for pre-surgical planning purposes, to monitor vessel stenosis or other conditions identified on previous CTA, MRA or non-invasive duplex ultrasound, or to confirm potential abnormality seen on ultrasound or MR scanning [9], [18]–[19]. As scanner capability has expanded, CTA protocols have evolved with the intention of improving the diagnostic value of a study. One such refinement has been to monitor contrast opacification of the aortic arch and to use this information to trigger the time of the imaging pass; this largely overcomes the variability in cardiac output between patients, and ensures that imaging takes place during optimized opacification of the vascular tree. Another refinement has been to add delayed phase imaging to a routine CTA protocol, with the expectation that additional information becomes available and that it may be useful for patient management.

Some reasons given for obtaining delayed phase imaging include identification of a patent internal carotid artery beyond a critical stenosis (pseudo-occlusion or “string” sign) [20], identification of active bleeding into an intracerebral hematoma [14] and evaluation of delayed filling of an intracranial aneurysm. While there are frequent requests for neurovascular CTA with delayed phase imaging, a literature search of PubMed (NLM) failed to identify any peer-reviewed publications that formally evaluate such a protocol.

The purpose of this study was to evaluate how often delayed images, obtained during neurovascular CTA, provide unique information relative to early phase imaging alone

## Material and Methods

### Ethics Statement

This retrospective study was approved by our institutional review board (Partners Human Research Committee, Partners Healthcare), waiving consent in accordance with the Health Insurance Portability and Accountability Act.

### Patient Population

A search of the electronic medical records (EMR) for patients scanned between January 1 and June 20, 2009 identified all neurovascular CTA studies that had delayed phase imaging. For these studies the radiology interpretation was reviewed to determine whether or not any information from the delayed phase images had been used to render the report. If the radiology report did not reference the delayed phase images it was taken that there was no unique or additional diagnostic information contained within it. In those cases where the delayed series was referenced, consensus review of the report was performed to determine if the information was truly unique to the delayed series, or if it was already present on the early phase images and thus merely confirmatory in nature. In those cases where the delayed series information contained in the report was potentially unique, the whole study was re-interpreted in PACS to confirm the uniqueness of the information.

### CT Imaging

The CTA imaging protocol consisted of a non-contrast head CT (NCCT) followed by a bolus-triggered CTA scanned from the skull vertex to the aortic arch. Scanning was performed on 64- or 16-slice multi-detector row CT scanners (LightSpeed VCT or LightSpeed Pro16, GE Medical Systems, Waukesha, WI, and Somatom Definition or Somatom Sensation Cardiac 64, Siemens Healthcare, Flanders, NJ). Patients were placed supine, head first in the scanner. An 18-gauge cannula was placed into a left or right antecubital vein and maintained with heparinized saline flush. Once the NCCT was complete the patient proceeded to the angiographic phase of the study. A single axial scan was acquired through the aortic root and a region of interest (ROI) placed in the ascending aorta for use with a manufacturer specific bolus tracking method that allows consistent timing of CTA imaging with respect to an individual patient's contrast hemodynamics. Contrast material (Isovue 370; Bracco Diagnostic Inc. Princeton, NJ) was injected in three phases; first, 60 ml at 4 ml/s, immediately followed by 15 ml at 2 ml/s, and finally by a 40 ml saline chase. The change in Hounsfield Units (HU) in the aortic ROI was monitored during injection; CTA imaging was triggered 10 seconds after this change exceeded 75 Hounsfield Units (HU). On the 64-slice scanners, the scanning parameters were either 120 or 140 kVp x-ray tube voltage, automatic tube current modulation, 64×0.6 mm detector configuration, 0.5 second gantry rotation time, and a 0.52 (GE) or 0.9 (Siemens) pitch factor. On the 16-slice scanner the scanning parameters were either 120 or 140 kVp x-ray tube voltage, automatic tube current modulation, 16×0.6 mm detector configuration, 0.5 second gantry rotation time and a 0.94 pitch factor. Images were reconstructed using a 512×512 matrix and the manufacturer-recommended reconstruction kernel for head scans. For scans obtained on the GE scanners, 1.25 mm images were reconstructed at 0.625 mm intervals; for those obtained on the Siemens scanners, 1.00 mm images were reconstructed at 0.6 mm intervals. Delayed phase imaging was acquired at a variable time after the initial phase.

### Data analysis

All CTA images were reviewed on our institutional PACS system (IMPAX 5.3, Agfa HealthCare Corp., Greenville, SC). The institutional EMR was consulted for the radiology reports in all patients and for clinical information in those patients with delayed phase imaging. Descriptive and statistical analyses were performed using SPSS version 20 (IBM Corp. Armonk, NY).

## Results

There were 1725 consecutive CTA studies performed during the search period; 645 had delayed phase imaging (645/1725; 37%). In the cohort with delayed phase imaging, there were 324 men (median age 67 years; range 20–92 years) and 310 women (median age 67; range 23–96 years). Six men and three women had 2 studies. One woman had 3 studies. The indications for the studies are provided in Table 1. The median elapsed time from completion of the first phase to the start of the delayed phase was 2.4 minutes, with an inter-quartile range of 2.2–2.8 minutes. In 15 studies, delayed imaging was obtained more than 6 minutes after the initial phase.

**Table 1:** Indications for 645 CTA Studies with Delayed Imaging.

	No.	%
Acute Stroke or TIA Evaluation	319	49.5
Carotid or Vertebral Artery Disease	132	20.5
Syncope; Weakness; Headache; dizziness; AMS	91	14.1
Aneurysm or AVM Evaluation	41	6.4
Evaluation of Trauma	29	4.5
Pre-Surgical Tumor Evaluation	13	2.0
Pre-Surgical Vessel Stenosis Evaluation	11	1.7
Evaluation for Potential Sinus Thrombosis	9	1.4

TIA  =  Transient Ischemic Attack; AMS  =  Altered Mental Status.

AVM  =  Arterio-Venous Malformation.

Fifty nine studies (59/645: 9.1%) had reports that specifically included observations obtained from the delayed phase images. There were 13 cases with hemorrhage, of which 4 showed progression on delayed images. Of the remaining 46 cases, 28 had occlusion of a vessel that did not reconstitute on the delayed images, 6 had occlusion of a vessel that did reconstitute on the delayed images, 7 had a string sign which was unchanged on the delayed views and 5 had no abnormal findings. In 41 cases (41/59: 69.5%) these observations provided information confirming findings that were present on the initial phase of imaging. In 7 cases (7/59: 11.9%), these observations identified unique additional information not present on the initial phase images. In the remaining 11 cases (11/59: 18.6%), there was uncertainty whether or not the observations provided unique information and these cases underwent consensus review. Three did contain unique, additional information, and 8 did not. Thus, of the whole cohort, there were 635 cases without (635/645; 98.45%) and 10 cases with (10/645; 1.55%) unique, additional information present on the delayed images.

**Figure 1:**
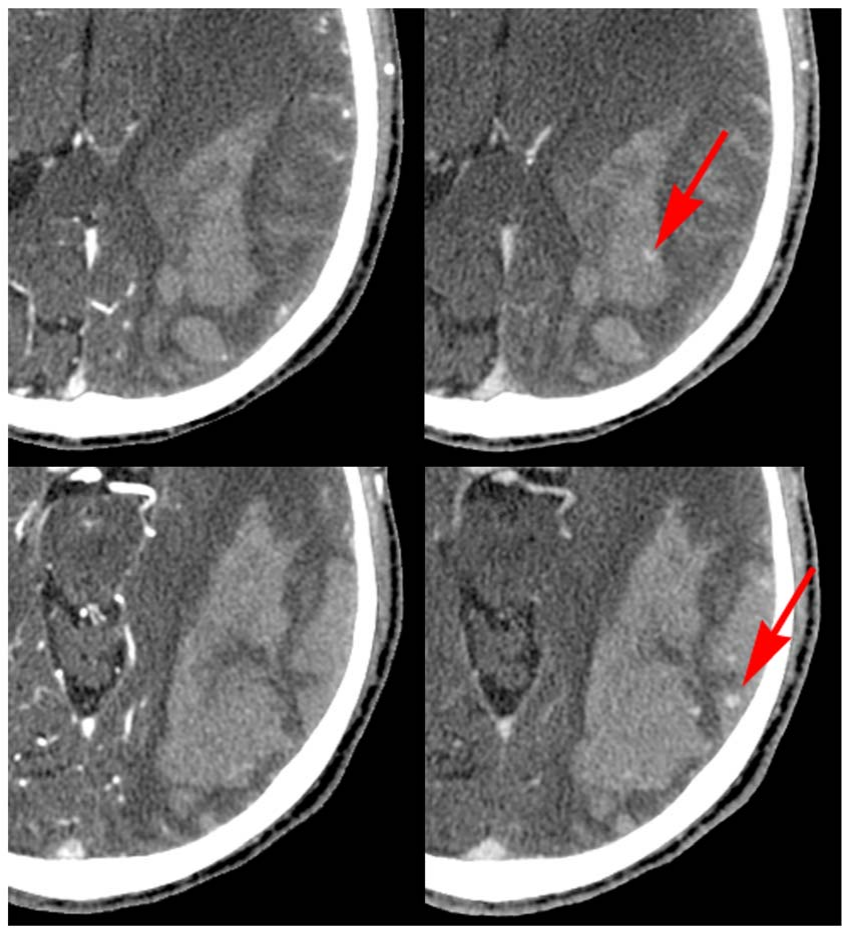
Example of active bleeding into a hematoma. 88 year old male with hypertension and acute stroke-like symptoms. CTA with delayed phase imaging shows a large hematoma in a left temporal/parietal location. Two axial levels are presented; on the left, images are from the initial phase imaging, on the right from the delayed phase. The red arrows indicate development of active extravasation seen only on the delayed views. The size of the hematoma was much larger than 80 ml and surgery was not performed, the presence of active bleeding did not change this patient's management decisions.

**Figure 2:**
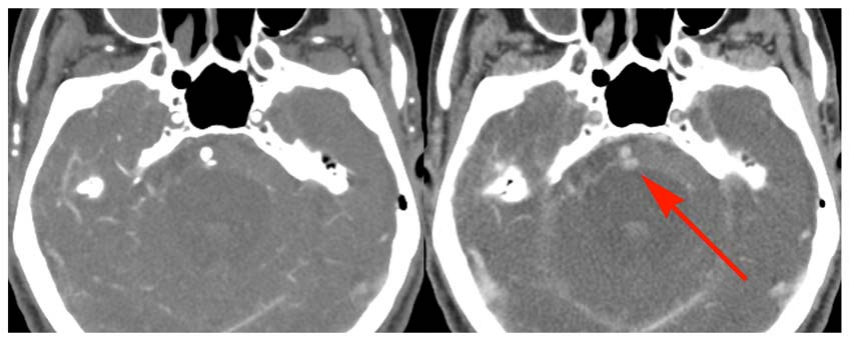
Example of active bleeding from a ruptured aneurysm. 86 year old male with two day history of lethargy with progressive stroke like symptoms. Early and delayed phase CTA images show subarachnoid hemorrhage with a 2(red arrow), consistent with active bleeding. Because of the patient's poor prognosis, and in adherence with his wishes, no intervention was performed. The presence of active bleeding on the delayed views did not change this management decision.

**Figure 3:**
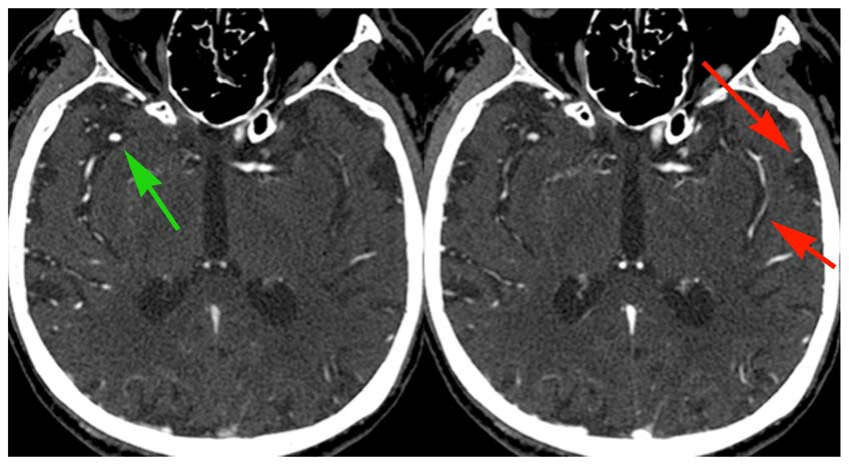
Example of delayed collateral vessels filling beyond a proximal occlusion. 83 year old male with atrial fibrillation and acute stroke. Early phase images show occlusion of the proximal M1 segment of the left MCA and robust opacification of the anterior division of the right MCA (green arrow). Delayed phase images show opacification of several pial vessels over the left temporal convexity and in the left Sylvian fissure (red arrows), delayed because of pial-pial collateral flow beyond the occlusion. Note that normal vessels demonstrate reduced opacification compared with the initial phase images (green arrow); this is due to decreased contrast concentration as a result of recirculation of the initial bolus. This CTA was obtained after MRI demonstrated the extent of MCA territory infarction; no management decisions were changed as a result of the delayed views.

Of the 10 cases with unequivocally unique, additional information on the delayed images, 3 had active bleeding into a cerebral hematoma (Fig. 1), and 1 had active bleeding around a left AICA aneurysm (Fig. 2). Three demonstrated delayed filling of the distal internal carotid artery from ophthalmic artery collateralization. Two demonstrated delayed filling of pial vessels distal to a proximal MCA occlusion (Fig. 3), and one demonstrated retrograde filling of a vertebral artery due to subclavian steal.

The 10 cases that we identified in our cohort of 645 patients (10/645: 1.55%) represent a sample estimate of the proportion of unique findings that would be found if the whole population of patients with delayed imaging on neurovascular CTA had been assessed. The 95% confidence limits for this estimate are 1.55±0.95%, representing no fewer than 4 and potentially up to 17 cases with unique information on the delayed views, if different, independent cohorts of 645 patients were to be studied.

## Discussion

This retrospective study has provided evidence that a delayed phase image series, obtained after the initial pass of a neurovascular CTA, may be useful in identifying active intracranial bleeding, collateral flow around a proximal occlusion or retrograde flow in subclavian steal syndromes. On the other hand, delayed imaging performed for pre-surgical planning purposes, to distinguish between pseudo-occlusion (“string” sign) and true occlusion of an internal carotid beyond a critical stenosis, did not provide unique discriminatory information in any case; if a string of contrast was seen in the internal carotid artery on the delayed views it was always present on the earlier phase. While objective evaluation of the impact of delayed view unique information on patient management is not available in this study, subjective examination of the medical records of those patients with unique information on the delayed views did not reveal any major deviation from the usual standard-of-care for their diagnosis. Formal evaluation to identify whether or not management is changed by the presence of unique information on the delayed views would require a randomized, prospective study.

The timing and indication for surgical intervention in the treatment of ICH remains controversial. Surgical intervention of supratentorial bleeds greater than 80 ml or less than 10 ml does not improve outcome versus conservative treatment; in these circumstances there appears to be little value in obtaining delayed imaging as, even if there is active bleeding, surgery would likely not be performed [21]. Nevertheless, as there is minimal additional risk to the patient, acquisition of a delayed series through the hematoma alone would provide definitive indication of active bleeding, which could be factored into patient management. However, if active bleeding has already been identified by the presence of a positive spot sign on the early phase images, delayed phase imaging becomes unnecessary as it would not add unique information [14]. As a result of the present study, our current CTA protocol for assessment of acute ICH explicitly includes delayed phase imaging only if a hematoma is identified on the non-contrast or early phase images, and then only through the hematoma. The determination of the need for delayed phase imaging is made by the supervising neuroradiologist at the time of scanning.

We did not find any case in our cohort in which a string sign was identified on delayed images alone. While this finding may be surprising it is not entirely unexpected; hemodynamic considerations and timing of the early phase images predict higher peak contrast concentration in the blood vessels than is seen on later images. This follows because on the delayed views, contrast re-distribution in the body has resulted in decreased opacification of all vessels, making a small residual lumen less visible than that seen on the early views. This is different to the expected findings that would result from catheter arteriography (DSA). In one reported technique, the conventional contrast injection at a rate of 8–11 ml/sec for one second into the distal common carotid artery was modified so that 16 ml of contrast were injected at 4 ml/sec for 4 seconds. The prolonged bolus and increased volume of contrast maximizes visibility of antegrade flow into a severely obstructed, yet incompletely occluded internal carotid artery, among other benefits [22]. Such a protocol does not translate to contemporary neurovascular CTA. As the present study shows, adding delayed imaging to neurovascular CTA does not reproduce the benefit of the modified DSA protocol. In an ROC analysis of neurovascular CTA, Lev et al. showed that a single pass CTA was highly accurate (85–95%) at distinguishing pseudo-occlusion from complete carotid occlusion, further supporting our findings that delayed views are unnecessary for identification of pseudo-occlusion [23].

Under some circumstances delayed views may indeed provide unique information, but this information does not necessarily alter patient care. An example is collateral opacification of a distal ICA in the presence of proximal occlusion. Vessels with normal antegrade flow are seen on the early phase, but due to the longer collateral pathway around a proximal occlusion, contrast may not reach a patent, distal vessel until later, which is thus better visualized on the delayed views. It should be noted that at the later time point, contrast may have already washed out from normally patent vasculature. Another potential source of misinterpretation of patency on delayed imaging is opacification of vasa-vasora; in cases of acute or recent subacute carotid occlusion, these small vessels may become inflamed and engorged, mimicking a “string” sign. Careful evaluation of the location of the contrast on the highest resolution images is necessary to avoid this pitfall.

In patients with compromised cardiac output, for instance due to atrial fibrillation or ischemic cardiomyopathy, both conditions that are encountered with some regularity in stroke patients, there may be a delay in contrast reaching the cervical and intracranial vasculature such that it is not visualized on conventionally timed images. In such cases, better visualization may be seen on the delayed images. However, most current protocols take account of this situation by monitoring contrast density in the aorta and timing the CTA to compensate for reduced cardiac output, substantially reducing the incidence of poor vessel opacification due to this particular situation.

One limitation of the present work is that the retrospective nature of our study means that any consultation between the referring physician and the interpreting neuroradiologist that took place at the time of imaging is lost. Addressing this would require a prospective study, which could be part of prospective evaluation of management decisions in different patient cohorts. Another limitation is that in crafting our analysis we assumed that unique information present on the delayed views would always be mentioned in the radiology report, and conversely, that absence of mention of findings on delayed phase images meant that there was no unique information contained within them. We believe that this approach was justified by clinical practice that dictates that no test should be performed if it is neither interpreted nor used in patient management. Another potential limitation is that we did not sample a sufficient number of studies. However, our confidence limits are narrow and we believe that the sample size does provide sufficient confidence in the results.

In summary, delayed phase imaging in neurovascular CTA may be useful in patients with acute ICH, as the presence of active bleeding potentially alters management by indicating the need for surgical evacuation of the clot. In the patients without hemorrhage, the value of delayed phase imaging is less obvious. Neither the ASA-AHA guideline on the management of patients with extracranial carotid and vertebral artery disease, nor the ACR-ASNR practice guidelines for the performance of cervicocerebral CTA include any recommendations for delayed phase imaging [24], [25]. In light of increasing scrutiny concerning appropriateness criteria for imaging in general, and cardiovascular CT in particular [26], [27], we make the following recommendations:

1). Obtaining delayed images during a neurovascular CTA, irrespective of indication, should be an active decision made by the supervising radiologist at the time of study acquisition, and not simply the default or routine protocol. 2). In the presence of hematoma, delayed imaging through the region of hemorrhage only may be useful to identify active bleeding and has minimal risk associated with the additional imaging. 3). If, at the time of reporting the study, it is deemed that delayed imaging may have been useful, but was not obtained, patient callback is appropriate. It is anticipated that such callback would be an infrequent occurrence as unique findings on delayed imaging in our cohort were rare.
